# 6,6-[Ethyl­enebis(sulfanediyl)]-2-(2-methoxy­ethyl)-1,2,3,4,5,6-hexa­hydro-1,5-methano-1*H*-azocino[4,3-*b*]indol-3-one

**DOI:** 10.1107/S1600536810015941

**Published:** 2010-05-08

**Authors:** Barış Tercan, Fatma Yüksel, Süleyman Patır, Tuncer Hökelek

**Affiliations:** a Department of Physics, Karabük University, 78050 Karabük, Turkey; b Department of Chemistry, Gebze High Technology Institute, 41400 Gebze, Kocaeli, Turkey; c Department of Chemistry Education, Faculty of Education, Hacettepe University, 06800 Beytepe, Ankara, Turkey; d Department of Physics, Hacettepe University, 06800 Beytepe, Ankara, Turkey

## Abstract

The title compound, C_19_H_22_N_2_O_2_S_2_, consists of a tetra­cyclic ring system containing an azocine skeleton with methoxy­ethyl and dithiol­ane groups as substituents. The benzene and five-membered *N*-heterocyclic rings are nearly coplanar, making a dihedral angle of 0.81 (12)°. The dithiol­ane ring adopts an envelope conformation. Inter­molecular N—H⋯O hydrogen-bonding and weak C—H⋯π inter­actions are present in the crystal structure.

## Related literature

For general background to the hexa­hydro-1,5-methano-azocino[4,3-*b*]indole core structure, a synthetic precursor for most of the penta­cyclic and tetra­cyclic indole alkaloids of biological inter­est, see: Hesse (2002 ▶); Bosch & Bonjoch (1988 ▶); Saxton (1983 ▶). For related structures, see: Hökelek *et al.* (2004 ▶, 2006 ▶, 2007 ▶); Tercan *et al.* (2010 ▶); Uludağ *et al.* (2006 ▶).
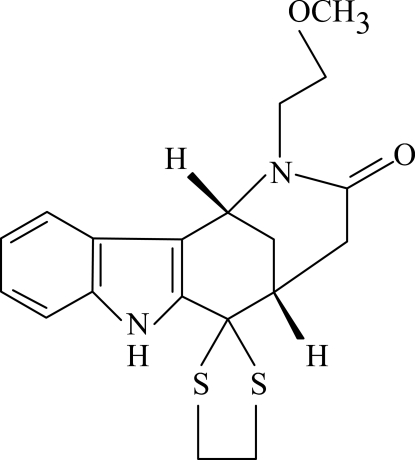


## Experimental

### 

#### Crystal data

C_19_H_22_N_2_O_2_S_2_
*M* *_r_* = 374.53
Monoclinic, 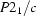

*a* = 11.2233 (3) Å
*b* = 15.4228 (5) Å
*c* = 12.3027 (4) Å
β = 121.267 (2)°
*V* = 1820.23 (10) Å^3^
*Z* = 4
Mo *K*α radiation
μ = 0.31 mm^−1^
*T* = 294 K
0.11 × 0.11 × 0.09 mm

#### Data collection

Bruker Kappa APEXII CCD area-detector diffractometer
Absorption correction: multi-scan (*SADABS*; Bruker, 2005 ▶) *T*_min_ = 0.85, *T*_max_ = 0.97
13979 measured reflections
3203 independent reflections
2765 reflections with *I* > 2σ(*I*)
*R*_int_ = 0.029

#### Refinement

*R*[*F* ^2^ > 2σ(*F* ^2^)] = 0.057
*wR*(*F* ^2^) = 0.159
*S* = 1.04
3203 reflections
279 parameters
1 restraint
H atoms treated by a mixture of independent and constrained refinement
Δρ_max_ = 1.10 e Å^−3^
Δρ_min_ = −0.56 e Å^−3^

### 

Data collection: *APEX2* (Bruker, 2007 ▶); cell refinement: *SAINT* (Bruker, 2007 ▶); data reduction: *SAINT*; program(s) used to solve structure: *SHELXS97* (Sheldrick, 2008 ▶); program(s) used to refine structure: *SHELXL97* (Sheldrick, 2008 ▶); molecular graphics: *ORTEP-3 for Windows* (Farrugia, 1997 ▶); software used to prepare material for publication: *WinGX* (Farrugia, 1999 ▶) and *PLATON* (Spek, 2009 ▶).

## Supplementary Material

Crystal structure: contains datablocks I, global. DOI: 10.1107/S1600536810015941/xu2755sup1.cif

Structure factors: contains datablocks I. DOI: 10.1107/S1600536810015941/xu2755Isup2.hkl

Additional supplementary materials:  crystallographic information; 3D view; checkCIF report

## Figures and Tables

**Table 1:** Hydrogen-bond geometry (Å, °) *Cg*1 is the centroid of the C7a/C8/C9/C10/C11/C11a ring.

*D*—H⋯*A*	*D*—H	H⋯*A*	*D*⋯*A*	*D*—H⋯*A*
N7—H7⋯O1^i^	0.82 (4)	2.06 (4)	2.832 (4)	157 (3)
C17—H17*A*⋯*Cg*1^ii^	0.96	2.89	3.545 (9)	127

Symmetry codes: (i) 
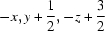
; (ii) 
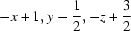
.

**Fig. 1.**
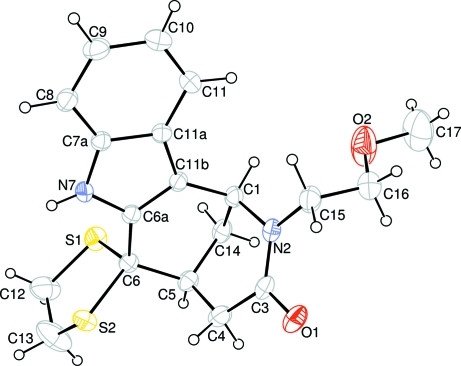
The molecular structure of the title molecule with the atom-numbering scheme. Displacement ellipsoids are drawn at the 50% probability level.

**Fig. 2.**
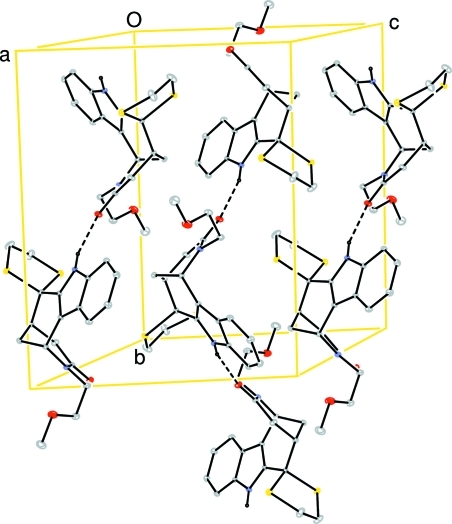
A partial packing diagram of the title compound. Hydrogen bonds are shown as dashed lines. H atoms not involved in hydrogen bonding have been omitted for clarity.

## supplementary crystallographic information

### Comment

The hexahydro-1,5-methano-azocino[4,3-b]indole core structure can be considered to be synthetic precursor for most of the pentacyclic and tetracyclic indole alkaloids of biological interests (Hesse, 2002; Bosch & Bonjoch, 1988; Saxton, 1983), such as akuminicine and uleine. Most of them have the pentacyclic ring system as a common element and include a large group of naturally occuring compounds such as strychnine, a consulvant poison, and uleine alkaloids.

The structures of tricyclic, tetracyclic and pentacyclic ring systems with different substituents of azocino[4,3-b]indole core have been determined, previously. These include *N*-(2-benzyloxyethyl)-4,7-dimethyl-6-(1,3-dithiolan- 2yl)-1,2,3,4,5,6-hexahydro-1,5-methano-2-azocino[4,3-b]indole-2-one, (II) (Hökelek *et al.*, 2004), 12-ethyl-2-methyl-6,6-ethylenedithio-1,2,3,4,5,6 -hexahydro-1,5-methano-2-azocino[4,3-b]indole-3-one, (III) (Uludağ *et al.*, 2006), 4-ethyl-6,6-ethylenedithio-2-(2-methoxymethyl)-7-methoxymethylene-2, 3,4,5,6,7-hexahydro-1,5-methano-1*H*-azocino[4,3-b]indole-3-one, (IV) (Hökelek *et al.*, 2006), 2-(2,2-dimethoxyethyl)-3-oxo-1,2,3,4,5,6 -hexahydro-1,5-methano-7*H*-azocino[4,3-b]indole, (V) (Hökelek *et al.*, 2007) and 2-ethyl-6,6-ethylenedisulfanediyl-7-methoxymethyl-1,2,3,4,5,6-hexahydro -1,5-methanoazocino[4,3-b]indol-3-one, (VI) (Tercan *et al.*, 2010). The present study was undertaken to ascertain the crystal structure of the title compound, (I).

The molecule of the title compound, (I), (Fig. 1) consists of a tetracyclic ring system containing an azocino skeleton with methoxyethyl and dithiolane groups as substituents at positions N2 and 6, respectively. The bonds N7—C6a [1.368 (4) Å] and N7—C7a [1.374 (4) Å] agree well with those in compounds (II) [1.392 (8) and 1.370 (8) Å], (IV) [1.393 (4) and 1.386 (5) Å], (V) [1.377 (3) and 1.376 (3) Å] and (VI) [1.398 (3) and 1.387 (3) Å]. The absolute configurations of C1 and C5 are S and S (Fig. 1). The S atoms of the dithiolane ring have electron-releasing properties, but the N atom at position 7 and the O atom attached to C3 have electron-withdrawing properties, leading to some changes in the bond lengths and angles of the carbazole skeleton.

An examination of the deviations from the least-squares planes through individual rings shows that rings A (C7a/C8/C9/C10/C11/C11a) and B (N7/C7a/C11a/C11b/C6a) are planar. They are also coplanar with a dihedral angle of A/B = 0.81 (12)°. Rings C (C1/C11b/C6a/C6/C5/C14), D (C1/N2/C3/C4/C5/C14) and E (C6/S1/S2/C12/C13) are, of course, not planar. Atom C14 deviates from the planes of F(C1/C5/C6/C6a/C11b) and G (C1/N2/C3/C4/C5) by 0.705 (4) Å and 0.737 (4) Å, respectively where the dihedral angle between planes of F and G is F/G = 69.64 (12)°. On the other hand, the dihedral angles between the plane of H (C1/C5/C14) and the planes of F and G are 54.18 (24)° and 56.47 (21)°, respectively. The conformation of ring E is an envelope, with atom C13 at the flap position, 0.583 (6) Å from the mean plane through the other four atoms.

In the crystal structure, intermolecular N—H···O hydrogen bonds (Table 1) link the molecules into chains nearly parallel to b-axis (Fig. 2), in which they may be effective in the stabilization of the structure. A weak C—H···π interaction also occurs (Table 1).

### Experimental

The title compound, (I), was prepared from 2,3-dichloro-5,6-dicyano-p -benzoquinone (0.68 g, 3.00 mmol) and N-(methoxyethyl)-(2,3,4,9-tetra -hydrospiro-[1H-carbazole-1,2'-(1,3)dithiolane]-2-yl)-2-acetamide (1.00 g, 2.68 mmol) in THF (35 ml). The mixture was stirred at room temperature for 4 h under nitrogen atmosphere, and then poured into sodium hydroxide solution (100 ml, 10%). After extraction with dichloromethane (50 ml), the organic layer was dried with Na_2_SO_4_ and the solvent was evaporated. The residue was purified by silicagel chromatography using triethylamine, acetone and ethyl acetate (7:25:75) and crystallized from ethyl acetate/diethyl ether (2:1) (yield; 0.87 g, 88%), m.p. 445 K.

### Crystal data

**Table:**

C_19_H_22_N_2_O_2_S_2_	*F*(000) = 792
*M**_r_* = 374.53	*D*_x_ = 1.367 Mg m^−^^3^
Monoclinic, *P*2_1_/*c*	Mo *K*α radiation, λ = 0.71073 Å
Hall symbol: -P 2ybc	Cell parameters from 6662 reflections
*a* = 11.2233 (3) Å	θ = 2.5–25.0°
*b* = 15.4228 (5) Å	µ = 0.31 mm^−^^1^
*c* = 12.3027 (4) Å	*T* = 294 K
β = 121.267 (2)°	Block, colourless
*V* = 1820.23 (10) Å^3^	0.11 × 0.11 × 0.09 mm
*Z* = 4	

### Data collection

**Table:**

Bruker Kappa APEXII CCD area-detector diffractometer	3203 independent reflections
Radiation source: fine-focus sealed tube	2765 reflections with *I* > 2σ(*I*)
graphite	*R*_int_ = 0.029
φ and ω scans	θ_max_ = 25.0°, θ_min_ = 2.1°
Absorption correction: multi-scan (*SADABS*; Bruker, 2005)	*h* = −13→13
*T*_min_ = 0.85, *T*_max_ = 0.97	*k* = −15→18
13979 measured reflections	*l* = −14→14

### Refinement

**Table:**

Refinement on *F*^2^	Primary atom site location: structure-invariant direct methods
Least-squares matrix: full	Secondary atom site location: difference Fourier map
*R*[*F*^2^ > 2σ(*F*^2^)] = 0.057	Hydrogen site location: inferred from neighbouring sites
*wR*(*F*^2^) = 0.159	H atoms treated by a mixture of independent and constrained refinement
*S* = 1.04	*w* = 1/[σ^2^(*F*_o_^2^) + (0.073*P*)^2^ + 2.6218*P*] where *P* = (*F*_o_^2^ + 2*F*_c_^2^)/3
3203 reflections	(Δ/σ)_max_ < 0.001
279 parameters	Δρ_max_ = 1.10 e Å^−^^3^
1 restraint	Δρ_min_ = −0.56 e Å^−^^3^

### Special details

**Table:**

Geometry. All esds (except the esd in the dihedral angle between two l.s. planes) are estimated using the full covariance matrix. The cell esds are taken into account individually in the estimation of esds in distances, angles and torsion angles; correlations between esds in cell parameters are only used when they are defined by crystal symmetry. An approximate (isotropic) treatment of cell esds is used for estimating esds involving l.s. planes.
Refinement. Refinement of *F*^2^ against ALL reflections. The weighted *R*-factor wR and goodness of fit *S* are based on *F*^2^, conventional *R*-factors *R* are based on *F*, with *F* set to zero for negative *F*^2^. The threshold expression of *F*^2^ > σ(*F*^2^) is used only for calculating *R*-factors(gt) etc. and is not relevant to the choice of reflections for refinement. *R*-factors based on *F*^2^ are statistically about twice as large as those based on *F*, and *R*- factors based on ALL data will be even larger.

### Fractional atomic coordinates and isotropic or equivalent isotropic displacement parameters (Å^2^)

**Table:**

	*x*	*y*	*z*	*U*_iso_*/*U*_eq_	
S1	−0.00713 (10)	0.85797 (6)	0.42981 (9)	0.0531 (3)	
S2	−0.17536 (8)	0.82283 (6)	0.54739 (8)	0.0505 (3)	
O1	−0.0128 (3)	0.51573 (16)	0.7174 (3)	0.0610 (7)	
O2	0.4150 (5)	0.4833 (2)	0.7731 (4)	0.1090 (14)	
C1	0.2094 (3)	0.6542 (2)	0.6617 (3)	0.0415 (7)	
H1	0.304 (4)	0.639 (2)	0.679 (3)	0.043 (9)*	
N2	0.1631 (3)	0.58379 (16)	0.7142 (3)	0.0431 (6)	
C3	0.0280 (4)	0.57123 (19)	0.6714 (3)	0.0443 (7)	
C4	−0.0805 (4)	0.6256 (3)	0.5630 (4)	0.0564 (9)	
H41	−0.142 (4)	0.587 (3)	0.502 (4)	0.068 (12)*	
H42	−0.141 (5)	0.653 (3)	0.592 (4)	0.077 (13)*	
C5	−0.0340 (3)	0.6908 (2)	0.4983 (3)	0.0430 (7)	
H5	−0.104 (3)	0.691 (2)	0.408 (3)	0.044 (9)*	
C6	−0.0220 (3)	0.7862 (2)	0.5439 (3)	0.0380 (7)	
C6A	0.1071 (3)	0.79611 (18)	0.6714 (3)	0.0339 (6)	
N7	0.1383 (3)	0.86796 (17)	0.7466 (2)	0.0366 (6)	
H7	0.087 (4)	0.909 (2)	0.735 (3)	0.042 (9)*	
C7A	0.2701 (3)	0.85823 (19)	0.8510 (3)	0.0356 (6)	
C8	0.3469 (3)	0.9135 (2)	0.9544 (3)	0.0447 (7)	
H8	0.307 (4)	0.965 (3)	0.963 (4)	0.065 (11)*	
C9	0.4779 (4)	0.8870 (3)	1.0455 (3)	0.0527 (9)	
H9	0.531 (4)	0.923 (2)	1.112 (4)	0.058 (11)*	
C10	0.5337 (4)	0.8091 (3)	1.0358 (4)	0.0577 (10)	
H10	0.617 (5)	0.798 (3)	1.094 (4)	0.070 (13)*	
C11	0.4588 (3)	0.7542 (2)	0.9344 (4)	0.0500 (8)	
H11	0.493 (4)	0.699 (3)	0.925 (3)	0.053 (10)*	
C11A	0.3232 (3)	0.77788 (19)	0.8398 (3)	0.0371 (7)	
C11B	0.2155 (3)	0.73953 (18)	0.7235 (3)	0.0362 (6)	
C12	−0.1673 (5)	0.9159 (3)	0.3688 (5)	0.0906 (17)	
H12A	−0.1475	0.9741	0.4036	0.109*	
H12B	−0.2121	0.9205	0.2771	0.109*	
C13	−0.2624 (5)	0.8740 (4)	0.3995 (6)	0.106 (2)	
H13A	−0.3178	0.8314	0.3344	0.127*	
H13B	−0.3256	0.9171	0.3991	0.127*	
C14	0.1063 (4)	0.6613 (2)	0.5194 (3)	0.0470 (8)	
H141	0.096 (3)	0.608 (2)	0.482 (3)	0.046 (9)*	
H142	0.145 (4)	0.704 (2)	0.483 (3)	0.056 (10)*	
C15	0.2666 (4)	0.5408 (2)	0.8317 (4)	0.0575 (9)	
H151	0.216 (6)	0.523 (4)	0.884 (5)	0.114 (18)*	
H152	0.346 (5)	0.582 (3)	0.883 (4)	0.076 (13)*	
C16	0.3309 (5)	0.4618 (3)	0.8156 (5)	0.0704 (11)	
H16A	0.3840	0.4319	0.8964	0.084*	
H16B	0.2586	0.4230	0.7556	0.084*	
C17	0.4859 (8)	0.4086 (4)	0.7611 (8)	0.126 (2)	
H17A	0.5444	0.4269	0.7298	0.189*	
H17B	0.4182	0.3679	0.7027	0.189*	
H17C	0.5419	0.3817	0.8427	0.189*	

### Atomic displacement parameters (Å^2^)

**Table:**

	*U*^11^	*U*^22^	*U*^33^	*U*^12^	*U*^13^	*U*^23^
S1	0.0634 (6)	0.0513 (5)	0.0516 (5)	0.0028 (4)	0.0346 (5)	0.0135 (4)
S2	0.0386 (4)	0.0590 (6)	0.0533 (5)	0.0041 (4)	0.0234 (4)	0.0035 (4)
O1	0.0779 (17)	0.0414 (13)	0.0728 (17)	−0.0145 (12)	0.0456 (15)	0.0025 (12)
O2	0.152 (3)	0.066 (2)	0.175 (4)	0.025 (2)	0.131 (3)	0.019 (2)
C1	0.0471 (18)	0.0336 (16)	0.0529 (19)	0.0004 (13)	0.0324 (16)	−0.0034 (13)
N2	0.0523 (16)	0.0296 (13)	0.0498 (15)	0.0011 (11)	0.0281 (13)	0.0007 (11)
C3	0.059 (2)	0.0292 (15)	0.0503 (18)	−0.0081 (14)	0.0327 (16)	−0.0075 (13)
C4	0.049 (2)	0.047 (2)	0.067 (2)	−0.0100 (17)	0.0265 (19)	0.0050 (18)
C5	0.0479 (18)	0.0403 (17)	0.0371 (17)	−0.0057 (14)	0.0196 (15)	−0.0025 (13)
C6	0.0407 (16)	0.0380 (16)	0.0379 (16)	−0.0017 (13)	0.0222 (14)	0.0014 (13)
C6A	0.0361 (14)	0.0338 (15)	0.0364 (15)	−0.0018 (12)	0.0220 (13)	−0.0006 (12)
N7	0.0375 (13)	0.0321 (13)	0.0402 (14)	0.0028 (11)	0.0200 (12)	−0.0024 (11)
C7A	0.0358 (15)	0.0349 (15)	0.0410 (16)	−0.0031 (12)	0.0234 (13)	−0.0005 (12)
C8	0.0470 (18)	0.0393 (17)	0.0503 (19)	−0.0047 (14)	0.0270 (16)	−0.0069 (14)
C9	0.0441 (18)	0.060 (2)	0.0455 (19)	−0.0104 (17)	0.0168 (16)	−0.0123 (17)
C10	0.0369 (18)	0.065 (2)	0.054 (2)	0.0004 (17)	0.0109 (17)	−0.0014 (18)
C11	0.0393 (17)	0.0452 (19)	0.061 (2)	0.0052 (15)	0.0234 (16)	0.0017 (16)
C11A	0.0349 (14)	0.0364 (15)	0.0432 (16)	−0.0015 (12)	0.0225 (13)	−0.0001 (13)
C11B	0.0383 (15)	0.0312 (14)	0.0436 (16)	−0.0016 (12)	0.0245 (13)	−0.0015 (12)
C12	0.056 (2)	0.091 (3)	0.100 (4)	0.008 (2)	0.023 (2)	0.050 (3)
C13	0.076 (3)	0.148 (5)	0.099 (4)	0.050 (3)	0.049 (3)	0.062 (4)
C14	0.065 (2)	0.0384 (18)	0.0494 (19)	−0.0033 (16)	0.0383 (18)	−0.0078 (15)
C15	0.063 (2)	0.0424 (19)	0.054 (2)	0.0027 (17)	0.0210 (19)	0.0035 (16)
C16	0.071 (3)	0.053 (2)	0.085 (3)	0.007 (2)	0.039 (2)	0.007 (2)
C17	0.165 (6)	0.097 (4)	0.188 (7)	0.030 (4)	0.141 (6)	0.002 (4)

### Geometric parameters (Å, °)

**Table:**

S1—C6	1.862 (3)	C9—C10	1.388 (5)
S1—C12	1.787 (5)	C9—H9	0.91 (4)
S2—C6	1.833 (3)	C10—C11	1.374 (5)
S2—C13	1.744 (5)	C10—H10	0.85 (4)
O1—C3	1.238 (4)	C11—C11A	1.401 (4)
O2—C16	1.337 (5)	C11—H11	0.96 (4)
O2—C17	1.450 (6)	C11A—C11B	1.437 (4)
C1—H1	0.99 (3)	C11B—C6A	1.358 (4)
N2—C1	1.487 (4)	C11B—C1	1.503 (4)
N2—C3	1.337 (4)	C12—C13	1.456 (7)
N2—C15	1.462 (5)	C12—H12A	0.9700
C4—C3	1.510 (5)	C12—H12B	0.9700
C4—H41	0.92 (4)	C13—H13A	0.9700
C4—H42	1.01 (5)	C13—H13B	0.9700
C5—C4	1.531 (5)	C14—C1	1.521 (5)
C5—C14	1.525 (5)	C14—H141	0.92 (4)
C5—H5	0.97 (3)	C14—H142	1.01 (4)
C6—C5	1.556 (4)	C15—C16	1.480 (6)
C6A—C6	1.492 (4)	C15—H152	1.00 (5)
N7—C6A	1.368 (4)	C15—H151	1.09 (6)
N7—C7A	1.374 (4)	C16—H16A	0.9700
N7—H7	0.81 (4)	C16—H16B	0.9700
C7A—C8	1.397 (4)	C17—H17A	0.9600
C7A—C11A	1.412 (4)	C17—H17B	0.9600
C8—C9	1.369 (5)	C17—H17C	0.9600
C8—H8	0.95 (4)		
			
C12—S1—C6	98.61 (18)	C9—C10—H10	117 (3)
C13—S2—C6	98.0 (2)	C11—C10—C9	121.4 (3)
C16—O2—C17	112.3 (4)	C11—C10—H10	121 (3)
N2—C1—C11B	110.8 (2)	C10—C11—C11A	118.8 (3)
N2—C1—C14	108.9 (3)	C10—C11—H11	124 (2)
N2—C1—H1	108.1 (19)	C11A—C11—H11	117 (2)
C11B—C1—C14	109.1 (3)	C7A—C11A—C11B	106.2 (2)
C11B—C1—H1	109.3 (19)	C11—C11A—C7A	118.6 (3)
C14—C1—H1	110.7 (19)	C11—C11A—C11B	135.2 (3)
C3—N2—C15	118.7 (3)	C6A—C11B—C1	122.0 (3)
C3—N2—C1	121.0 (3)	C6A—C11B—C11A	106.9 (3)
C15—N2—C1	118.9 (3)	C11A—C11B—C1	131.2 (3)
O1—C3—N2	122.3 (3)	S1—C12—H12A	109.1
O1—C3—C4	117.9 (3)	S1—C12—H12B	109.1
N2—C3—C4	119.8 (3)	C13—C12—S1	112.5 (3)
C3—C4—C5	119.2 (3)	C13—C12—H12A	109.1
C3—C4—H41	106 (3)	C13—C12—H12B	109.1
C3—C4—H42	107 (3)	H12A—C12—H12B	107.8
C5—C4—H41	108 (3)	C12—C13—S2	112.5 (4)
C5—C4—H42	113 (3)	C12—C13—H13A	109.1
H41—C4—H42	101 (4)	C12—C13—H13B	109.1
C4—C5—C6	114.9 (3)	S2—C13—H13A	109.1
C4—C5—H5	108 (2)	S2—C13—H13B	109.1
C6—C5—H5	106 (2)	H13A—C13—H13B	107.8
C14—C5—C4	108.4 (3)	C1—C14—C5	108.6 (3)
C14—C5—C6	109.4 (3)	C1—C14—H141	109 (2)
C14—C5—H5	110.6 (19)	C1—C14—H142	108 (2)
C5—C6—S1	108.4 (2)	C5—C14—H141	110 (2)
C5—C6—S2	113.2 (2)	C5—C14—H142	112 (2)
C6A—C6—C5	109.3 (3)	H141—C14—H142	109 (3)
C6A—C6—S1	108.3 (2)	N2—C15—C16	115.7 (3)
C6A—C6—S2	110.8 (2)	N2—C15—H151	108 (3)
S2—C6—S1	106.67 (16)	N2—C15—H152	109 (2)
N7—C6A—C6	124.2 (3)	C16—C15—H151	108 (3)
C11B—C6A—N7	110.5 (3)	C16—C15—H152	105 (2)
C11B—C6A—C6	125.0 (3)	H152—C15—H151	111 (4)
C6A—N7—C7A	108.5 (3)	O2—C16—C15	109.9 (4)
C6A—N7—H7	127 (2)	O2—C16—H16A	109.7
C7A—N7—H7	124 (2)	O2—C16—H16B	109.7
N7—C7A—C8	129.8 (3)	C15—C16—H16A	109.7
N7—C7A—C11A	108.0 (3)	C15—C16—H16B	109.7
C8—C7A—C11A	122.1 (3)	H16A—C16—H16B	108.2
C7A—C8—H8	121 (2)	O2—C17—H17A	109.5
C9—C8—C7A	117.1 (3)	O2—C17—H17B	109.5
C9—C8—H8	122 (2)	O2—C17—H17C	109.5
C8—C9—C10	121.9 (3)	H17A—C17—H17B	109.5
C8—C9—H9	118 (2)	H17A—C17—H17C	109.5
C10—C9—H9	120 (2)	H17B—C17—H17C	109.5
			
C12—S1—C6—S2	−7.3 (3)	C11B—C6A—C6—S1	−101.0 (3)
C12—S1—C6—C5	114.9 (3)	C11B—C6A—C6—S2	142.3 (3)
C12—S1—C6—C6A	−126.6 (3)	C11B—C6A—C6—C5	16.8 (4)
C6—S1—C12—C13	−15.1 (5)	C7A—N7—C6A—C6	−174.4 (3)
C13—S2—C6—C6A	140.4 (3)	C7A—N7—C6A—C11B	−0.3 (3)
C13—S2—C6—C5	−96.3 (3)	C6A—N7—C7A—C8	−179.4 (3)
C13—S2—C6—S1	22.8 (3)	C6A—N7—C7A—C11A	0.4 (3)
C6—S2—C13—C12	−35.3 (5)	N7—C7A—C8—C9	−179.6 (3)
C17—O2—C16—C15	177.4 (5)	C11A—C7A—C8—C9	0.6 (5)
C3—N2—C1—C11B	82.0 (4)	N7—C7A—C11A—C11	178.5 (3)
C3—N2—C1—C14	−38.0 (4)	N7—C7A—C11A—C11B	−0.4 (3)
C15—N2—C1—C11B	−84.5 (3)	C8—C7A—C11A—C11	−1.7 (4)
C15—N2—C1—C14	155.6 (3)	C8—C7A—C11A—C11B	179.4 (3)
C1—N2—C3—O1	−176.6 (3)	C7A—C8—C9—C10	0.7 (5)
C1—N2—C3—C4	3.6 (5)	C8—C9—C10—C11	−0.8 (6)
C15—N2—C3—O1	−10.1 (5)	C9—C10—C11—C11A	−0.4 (6)
C15—N2—C3—C4	170.1 (3)	C10—C11—C11A—C7A	1.6 (5)
C1—N2—C15—C16	−92.1 (4)	C10—C11—C11A—C11B	−179.9 (3)
C3—N2—C15—C16	101.1 (4)	C7A—C11A—C11B—C1	−178.8 (3)
C5—C4—C3—O1	−176.1 (3)	C7A—C11A—C11B—C6A	0.3 (3)
C5—C4—C3—N2	3.7 (5)	C11—C11A—C11B—C1	2.5 (6)
C6—C5—C4—C3	−98.9 (4)	C11—C11A—C11B—C6A	−178.4 (4)
C14—C5—C4—C3	23.9 (5)	C6A—C11B—C1—N2	−94.1 (3)
C4—C5—C14—C1	−57.4 (4)	C6A—C11B—C1—C14	25.7 (4)
C6—C5—C14—C1	68.7 (3)	C11A—C11B—C1—N2	84.9 (4)
S1—C6—C5—C4	−166.5 (2)	C11A—C11B—C1—C14	−155.3 (3)
S1—C6—C5—C14	71.2 (3)	C1—C11B—C6A—N7	179.2 (3)
S2—C6—C5—C4	−48.4 (3)	C11A—C11B—C6A—N7	0.0 (3)
S2—C6—C5—C14	−170.6 (2)	C1—C11B—C6A—C6	−6.7 (4)
C6A—C6—C5—C4	75.7 (3)	C11A—C11B—C6A—C6	174.1 (3)
C6A—C6—C5—C14	−46.6 (3)	S1—C12—C13—S2	34.0 (7)
N7—C6A—C6—S1	72.2 (3)	C5—C14—C1—N2	65.4 (3)
N7—C6A—C6—S2	−44.4 (3)	C5—C14—C1—C11B	−55.6 (3)
N7—C6A—C6—C5	−169.9 (3)	N2—C15—C16—O2	70.2 (5)

### Hydrogen-bond geometry (Å, °)

**Table:**

Cg1 is the centroid of the C7a/C8/C9/C10/C11/C11a ring.

**Table:**

*D*—H···*A*	*D*—H	H···*A*	*D*···*A*	*D*—H···*A*
N7—H7···O1^i^	0.82 (4)	2.06 (4)	2.832 (4)	157 (3)
C17—H17A···Cg1^ii^	0.96	2.89	3.545 (9)	127

Symmetry codes: (i) −*x*, *y*+1/2, −*z*+3/2; (ii) −*x*+1, *y*−1/2, −*z*+3/2.
